# A Model for Improving Diet Quality within Child Nutrition Programs: The U.S. Army’s Child and Youth Services Healthy Menu Initiative

**DOI:** 10.3390/ijerph17082746

**Published:** 2020-04-16

**Authors:** Jennifer Hanson, Jillian Joyce, Denise Laursen, Paola Paez

**Affiliations:** 1 Department of Food, Nutrition, Dietetics and Health, Kansas State University, Manhattan, KS 66506, USA; 2 Department of Nutritional Sciences, Oklahoma State University, Stillwater, OK 74078, USA; jill.joyce@okstate.edu; 3 Department of the Army, Installation Management Command, G9-Child and Youth Services, Joint Base San Antonio-Fort Sam Houston, San Antonio, TX 78234, USA; denise.e.laursen.naf@mail.mil; 4 The Center for Food Safety in Child Nutrition Programs, Kansas State University, Manhattan, KS 66506, USA; paolap@ksu.edu

**Keywords:** child nutrition, healthy eating index, menu planning, food service

## Abstract

The U.S. Army’s Child, Youth, and School Services (CYS), which has the capacity to serve more than 70,000 meals/snacks per day, is a geographically dispersed system with facilities worldwide. This case report is a description and evaluation of the implementation of a major food program initiative within the CYS system. In collaboration with Kansas State University, the Healthy Menu Initiative was established to standardize the system’s menus, reflect the guidance contained within the 2015–2020 Dietary Guidelines for Americans, and take into account the Child and Adult Care Food Program regulations that went into effect on 1 October 2017. Food storage space, food service equipment, product availability, food safety considerations, and staff shortages have all proven to be challenges in the development and implementation of the menus. Participant acceptance has been an issue in some instances, and special diet requirements add to the workload of the staff. To overcome these challenges, input was solicited from CYS management, care providers, food service staff, and participant families, as well as participants themselves. Taste testing and classroom cooking activities have been developed to increase acceptance, and over 500 CYS food program staff have attended in-depth training sessions to support the initiative. Overall, the initiative has been well received, and there has been an improvement in the diet quality of the foods served within the program. This improvement is noteworthy, as optimal growth and development during childhood and adolescence are reliant on sound nutrition.

## 1. Introduction

The U.S. Army’s Child, Youth, and School Services (CYS) is a geographically dispersed child services system, tasked with supporting the Army’s mission readiness through the provision of quality child and youth services. The system operates facilities in the U.S., Europe, and throughout the Pacific Region. CYS offers a variety of programs that serve children between 6 weeks and 18 years of age. Hours of operation vary, but include early morning and evening hours, weekends, and overnight care at some locations. Most of the programs offer meals and/or snacks as part of the services provided. These programs include child development centers, school age centers, and youth centers. 

The CYS food program encompasses the operational facets related to the provision of food throughout CYS. Like the overall system, the food program operates worldwide and provides snacks/meals at over 350 child services facilities located on 72 installations. In total, the CYS program serves up to 70,000 meals/snacks per day [1]. 

Although the authority and responsibility for the provision of meals and snacks fall to the directors of each facility, technical support and assistance is available from the CYS Installation Management Command (IMCOM) staff located at IMOCM Headquarters, Joint Base San Antonio-Fort Sam Houston, TX. The CYS subject matter expert provides guidance in regard to nutrition, food service practices, food safety issues, and supply concerns. While the number of food program employees differ by location, there are over 450 kitchen staff positions, some of which are part-time positions. The Army installations vary by size, and large installations have the option of employing a nutrition specialist to take on the administration of the food and nutrition program at their location. Within the installations, the child services facilities also vary by size, with some small facilities providing services for approximately 25 children, while other larger facilities serve over 300 children.

The CYS food program is required by regulation (AR 608-10) [2] to comply with the standards of the Child and Adult Care Food Program (CACFP). In April 2016, the CACFP meal standards were updated [3] to better align with the Dietary Guidelines for Americans (DGA) [4]. 

However, at the direction of the CYS Division Chief, an initiative to revolutionize the CYS food and nutrition program had already been set into motion. The initiative, herein referred to as the Healthy Menu Initiative, transformed the food program through the implementation of standardized, seasonal, nutritious, and cost-effective menus while meeting child nutrition best practices recommendations. Implementation of the initiative was undertaken in collaboration with Kansas State University and began with staff training and the testing of a pilot menu. 

The initiative reflects the guidance contained within the DGA and takes into account the CACFP rules and best practices that went into effect on 1 October 2017. The Healthy Menu Initiative continues to evolve in response to both new and ongoing demands. Despite a number of challenges, the initiative has been well received, and a comparison of pre-initiative menus with a current menu indicates an improvement in diet quality has occurred since implementation of the initiative began. 

This paper presents an account and evaluation of the implementation of a major food program initiative in a geographically dispersed child services system operating facilities worldwide. A discussion of the challenges encountered as well as the solutions put forth is included. Reflections in the context of the current literature are also included. The program is open to collaboration and sharing of best practices with organizations for the purpose of improving child nutrition. The program resources are available to all military services, and select materials are available to the public through a learning system operated by The Ohio State University [5]. 

## 2. Menu Development

Menu planning influences all other food service tasks and management functions, such as staffing, procurement, storage, and customer relations [6]. Prior to the Healthy Menu Initiative, there was not an Army-wide standard for CYS menus, and there was very little menu consistency between locations [1]. Instead, CYS menus were written and approved by a qualified professional at the local (i.e., installation) or regional (i.e., Europe) level. Therefore, menu development for the initiative began with the goal of creating standardized, seasonal, nutritious, and cost-effective menus for use in CYS programs Army-wide. 

**Figure 1:**
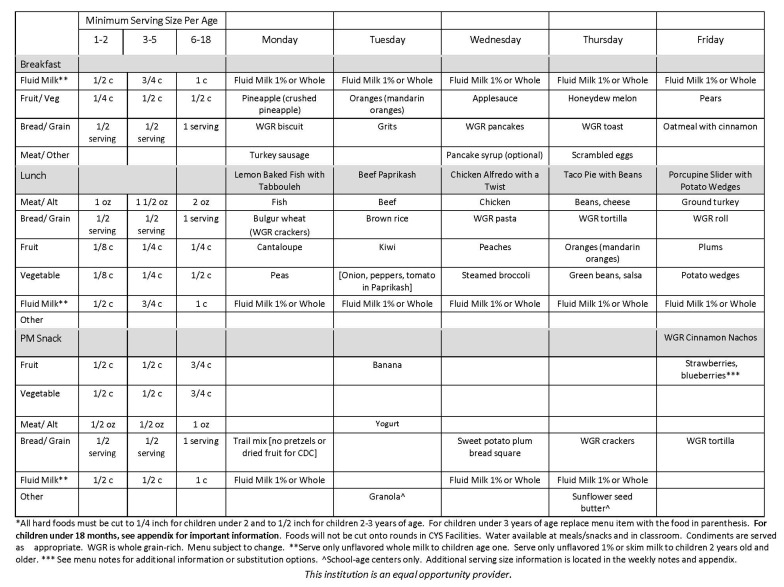
Sample week of the pilot menu for the Healthy Menu Initiative.

The initial phase included the creation and evaluation of a pilot menu. An eight-week cyclic menu format was selected to minimize the perception of redundancy. The menu included breakfast, lunch, and afternoon snack and was created on the basis of the CACFP meal patterns requirements. The food components (i.e., food groups) that made up the menu are listed in the sample week of the pilot menu depicted in Figure 1. The pilot menu was developed to be implemented in the fall of 2016 and winter of 2017. Consequently, the foods incorporated in the menu were those foods typically available during the cold weather months in most locations within the U.S. To assure agreement with the DGA recommendation for variety [4], a weekly checklist was developed to assure that an assortment of grains, fruits, vegetables, and protein sources were incorporated into the menus. As recommended by Wojcicki and Heyman [7], juice was not included in the menu. Processed meats (i.e., ham, sausage) were listed no more than once per week. Beans, eggs, and cheese were used to create one meatless lunch meal each week. Most entrees were menu items designed to be prepared from recipes. The majority of the recipes were U.S. Department of Agriculture (USDA) child nutrition recipes.

Five locations consisting of four installations within the Continental United States (CONUS) and all of the CYS facilities in Europe implemented the pilot menu. The CONUS installations included two large installations, each having more than five child services facilities, and two small installations, each having five or fewer facilities. Feedback was collected using a menu feedback data collection tool developed to measure preparation effort, preparation time, quality of the ingredients available, quality of the finished products, special equipment required for the preparations, special culinary skills required for the preparations, general comments, and overall feasibility. The data collection tool was customized for each week of the menu, and feedback was collected weekly. The tool along with examples of the feedback that was collected can be viewed in Supplement 1. While over 25 facilities provided feedback, not every facility provided feedback every week. 

Based on the pilot survey feedback, the eight-week cycle was modified to a five-week cycle. This change allowed centers to reduce the size of their food inventory and decreased the repertoire of skills needed to complete the cycle [6], while still maintaining minimal perception of redundancy. Pilot survey feedback also resulted in the elimination of some entrees due to low acceptance, impracticality, or issues surrounding the availability of ingredients. 

Using feedback from the pilot project, a new menu was developed for implementation in CYS Army-wide facilities during the spring of 2017. Since the CYS-wide rollout of the menu, three new sets of standardized, seasonal, nutritious, and cost-effective menus have been developed each year. Each set of menus contains an infant, child development center, school age center, and youth center version. Separate menus were developed for day care homes as an option that providers can use if they choose. A packed lunch menu with 10-days’ worth of field trip lunches was also developed. 

The menus continue to evolve in response to both new and ongoing demands, such as changes in product availability and stakeholder feedback. The program continues to solicit input from stakeholders including CYS management, care providers, food service staff, and participant families, as well as participants themselves. For example, CYS patrons can use the web-based Interactive Customer Evaluation (ICE) system to provide feedback regarding the menu program. Feedback has been gleaned from a variety of other sources as well, including staff training sessions and the Army Youth Leadership Forum [8]. 

The current meals reflect the guidance contained within the 2015–2020 DGA [4] and take into account the CACFP rules and best practices that went into effect on 1 October 2017 [3]. Per the new CACFP best practices guidelines [3], either a vegetable or a fruit is included as an afternoon snack each day, and a minimum of one serving from each of the five vegetable groups is provided each week. On days in which a fruit is served for the afternoon snack, either a vegetable is served at breakfast or two vegetables are served for lunch, rather than the typical one vegetable and one fruit serving for lunch. Because vegetable consumption is well below the recommended goals, while fruit consumption among preschool-aged children is relatively high [4], this practice shifts the day’s servings closer to the DGA goals set for the preschool age group. Due to the potential for nutrient imbalances [9], the CACFP option that allows a meat/meat alternative to be served in place of breakfast grains was not implemented.

## 3. Overcoming Challenges

Per the menu initiative, the use of raw ingredients and minimally processed foods is encouraged. The use of less processed ingredients may represent a food safety challenge, considering the necessary and additional steps to be incorporated to prevent cross-contamination and cross-contact for food allergies. The CYS food program uses the military specific Tri-Service Food Code [10]; this code establishes uniform military food safety standards for programs to follow. Knowledge and comprehension of the Tri-Service Food Code is essential for the programs to be able to follow the required food safety standards. 

To minimize the risk of foodborne illness, all recipes that contained eggs were modified to call for pasteurized eggs. In addition, a food safety round table was conducted to gather input from the nutrition specialists (*n* = 12) from an array of installations. As a function of their scope of responsibility, these nutrition specialists regularly interact with the kitchen staff at their respective installations. Based on a thematic analysis of the recorded session, a plan was developed to systematically incorporate food safety prompts into all recipes. For example, if a raw time and temperature control for safety (TCS) food is handled as an intermediate step in a recipe, the following statement is included in the instruction section of the recipe: “Thoroughly wash, rinse, and sanitize surfaces and equipment immediately after handling potentially hazardous [TCS] foods, and before proceeding to the next recipe step”.

To assist the food service staff in preparing meals, each set of menus contains additional notes, which list the appropriate serving sizes for each age category, specify substitutions for the prevention of food-related choking in younger age groups, and offer suggestions for non-available items. To minimize the risk of choking, CYS implements strict guidelines to prevent the serving of foods regarded as choking hazards. In addition, as the menu initiative was undertaken, a speech–language pathologist was consulted to provide advice on menu modification and substitutions for infants and young children. 

Each weekly set of menus is accompanied by a production calculator designed to assist the food service staff in calculating production quantities and scaling recipes. The food service staff enters the forecasted number of children in each age category, and the weekly production calculators compute the amount of each menu item needed. For recipes, the production calculators compute the total number of servings required and then scale the recipe ingredients based on the total number of servings needed. 

An extensive effort was made to provide CYS food program staff with the knowledge and skills necessary to implement the Healthy Menu Initiative. To date, over 500 CYS staff members (primarily cooks) have attended the four-day training sessions developed specifically to meet their needs. The age range of the training attendees spanned young adulthood (i.e., individuals 18–25 years of age) through later adulthood (i.e., individuals 60 years of age and older). Participants’ length of time working in food service ranged from under 1 year to more than 20 years. 

The training sessions were comprised of classroom lessons, as well as food preparation and other hands-on activities. A registered dietitian with a Ph.D. in nutrition and prior work experience within the military’s child services food program had overall responsibility for the training program. A research scientist with a Ph.D. and 13 years of experience in food safety and the food service industry was responsible for the food safety and hands-on training. Classroom topics included food safety, food allergies, nutrition, time management, and recipe conversions. Training attendees spent time in the kitchen practicing knife skills, preparing new recipes, monitoring and following food safety practices, and familiarizing themselves with different types of food service equipment. Resources provided by the Institute of Child Nutrition at the University of Mississippi were shared with the attendees. The attendees were also provided the opportunity to evaluate the recipes they prepared and to provide feedback regarding the training. Roughly, 30% of the training time was spent on nutrition, 30% was spent in preparing food, 20% was spent on food safety, and 20% was spent on food production management and recipe conversion.

## 4. Evaluation 

The Healthy Eating Index (HEI)-2015 [11] was used to assess the diet quality of a current CYS menu along with that of two corresponding menus that were in effect immediately prior to the implementation of the Healthy Menu Initiative. The HEI-2015 [11] is a tool that measures diet quality by assigning a numeric value representative of how well a given set of foods aligns with the 2015–2020 DGA [4]. Thirteen components (i.e., items) contribute to the total HEI-2015 score, with a total of 100 points possible. Higher scores indicate greater alignment with the DGA and thus higher diet quality [11]. The components include food groups as well as nutrients. Nine of the components correspond to measures of adequacy (i.e., total fruits, whole fruits, total vegetables, greens and beans, whole grains, diary, total protein foods, seafood and plant proteins, and fatty acids), where higher intake results in a higher score. Four of the components correspond to measures of moderation (refined grains, sodium, added sugars, and saturated fats), such that lower intake results in a higher score [11]. 

The menus selected for the pre-initiative evaluation included a menu from a large installation with more than 10 facilities and a menu from a small installation with 3 facilities. Both pre-initiative menus were approved by a registered dietitian, met the CACFP meal component requirements, and were in effect during the spring–summer of 2016. The corresponding post-initiative menu was in effect during the spring of 2019 (Supplement 2). 

For the purpose of this evaluation, the 3–5 years age category was used to determine food-serving sizes. Nutrient analysis was powered by the ESHA Research Nutrient Database©. To achieve accuracy and consistency in the entering of foods into the database, a codebook was developed for foods that commonly appeared on the menus (e.g., saltine crackers, brown rice, apples). Condiments, including salad dressing, spreads, and pancake syrup, were included in the analysis. For preparations containing more than one component such as pizza, CACFP-creditable recipes were used for the nutrient analysis. In such instances, the portion size for one or more components was sometimes greater than the minimum meal pattern requirement. For example, the turkey sandwich recipe met the meat/meat alternate minimum requirement, but provided three times the grain minimum requirement. For the entry of foods not based on a mixed dish recipe, the quantity of food entered was determined on the basis of the minimum serving amount needed to fulfil the CACFP requirement. To maintain consistency in the evaluation of the menus, the quantity of ready-to-eat breakfast cereals was determined using the portion size rule in effect beginning 1 October 2019 [3].

The ESHA output values for energy, monounsaturated fatty acids, polyunsaturated fatty acids, saturated fatty acids, added sugar, and sodium were used to calculate their corresponding HEI-2015 component scores. Values that were missing from the ESHA output were obtained from product labels or the USDA FoodData Central [12] database. The food-based component scores of the HEI-2015 were calculated by reviewing each menu and totaling the servings of each food component (e.g., legumes, refined gains, whole fruits) provided. Daily HEI-2015 scores were calculated by totaling each day’s component scores. 

Descriptive data were calculated for energy (kcals), HEI-2015 scores, and scores for each of the HEI-2015 components. Data were checked for normality and homogeneity of variance. Because energy values were non-normally distributed (Sharpiro–Wilk, *p* = 0.021) within the large-installation pre-initiative menu group, one-way ANOVA on ranks (i.e., Kruscal–Wallis) was used to compare daily mean energy. Mean HEI-2015 scores were compared using one-way ANOVA, as the assumptions of normality and homogeneity of variance were met for each of the menu groups. As a statistically significant difference in HEI-2015 was detected between the three menus as a whole, a Tukey post hoc test was run to determine which menus differed significantly. For each of the HEI-2015 components, a comparison was conducted to identify component score differences between the pre-initiative and the post-initiative groups. Because most of the HEI-2015 component scores were non-normally distributed within the two groups, Mann–Whitney U tests were used to detect differences. A significance level of *p* < 0.05 was used for all analyses. Statistical analysis was performed using SPSS analytic software (version 25, IBM Corporation, Armonk, NY, USA).

### HEI Evaluation Results

Results of the menu comparison are provided in Table 1. While the menus did not differ significantly with regard to energy (kcals) (*p* = 0.502), they did vary as a whole with regard to mean HEI-2015 score (*p* ≤ 0.001). Post hoc analysis revealed that the post-initiative menu mean HEI-2015 score was statistically higher than mean scores for both the small- and large-garrison pre-initiative menus (75.9 vs. 67.8 and 66.2, respectively). HEI-2015 scores for the two pre-initiative menus did not differ significantly from one another (*p* = 0.751). 

For all three menus, the maximum points for the dairy and whole-fruit components of the HEI-2015 were achieved every day. The maximum points possible were achieved for the refined-grain component on all but one day (*n* = 69), and the maximum points possible were attained for the total fruit component on all but two days (*n* = 68). Conversely, the maximal points for the whole-grain and fatty-acids components were each only achieved on one day. The occurrence of maximum HEI-2015 for all HEI-2015 components is provided in Table 2. 

Table 3 provides the results of the comparison of scores for each of the HEI-2015 components. Compared to the pre-initiative menus, the post-initiative menu achieved higher component scores for total vegetables (*p* < 0.001), fatty acids (*p* = 0.010) and greens and beans (*p* = 0.025).

## 5. Discussion and Lessons Learned 

Our evaluation indicates that with the implementation of the Healthy Menu Initiative, there has been an improvement in the diet quality of the U.S. Army’s CYS menus. As a result of the standardization that occurred as a part of the initiative, children throughout the CYS system have benefited from consistently higher quality menus regardless of their location. While dairy and whole-fruit offerings were optimal prior to the initiative, following the initiative there has been an improvement in total diet quality, fatty acid composition, and the offerings of vegetables. Although the fatty acid composition of the menus has improved significantly, the score for this component remains relatively low. Further improvements in diet quality can be achieved by increasing the ratio of healthier to less healthy fatty acids. 

Both pre-initiative and post-initiative menus received notably low scores for whole grains. Only whole-grain-rich products were used to fulfill the grain requirement on the post-initiative menus. Although these products met the CACFP standard for whole-grain-rich products, many were not 100% whole grain. In applying the HEI-2015, only products known to be 100% whole-grain (i.e., brown rice) received points. Although product availability may be an issue, facilities wishing to further improve the diet quality of the meals they serve should seek out 100% whole-grain products for their menus. 

Although a limitation of this evaluation is the fact that information regarding the actual consumption of foods is missing, prior research has revealed an association between the nutritional quality of the food consumed and that of the food served in childcare settings [13]. While it is beyond the scope of this current evaluation, future research is needed to assess the initiative’s impact on dietary intake. In addition to its role of assuring high-quality menus throughout CYS, the menu standardization that occurred as a part of the initiative has resulted in an overall saving of the countless person-hours that were once spent on developing and reviewing menus at an array of locations throughout CYS. However, standardization and implementation of the menus have led to the disappearance of some familiar foods (e.g., granola bars) and the introduction of other more novel foods (e.g., quinoa, lentils). Child nutrition managers cited “pressure to serve foods kids enjoy” as a common perceived barrier to improving diet quality [14], and the initiative menu changes were projected to create issues related to acceptance. To overcome the challenges associated with acceptance, input was solicited from CYS management and care providers, CYS food service staff, and participant families, as well as school-aged and youth center participants. Information about the initiative was shared during parent advisory board meetings and through local news outlets [15]. Taste-testing activities and classroom cooking activities have also been developed to increase acceptance and foster food preparation skills among CYS participants. These activities have been endorsed by mangers as a means to improve food choices [14] and they may play a role in increasing selection and consumption of healthy foods [16,17]. 

Food storage space, food service equipment, product availability, food safety considerations, and staff shortages have all proven to be challenges in the development and implementation of the menus. Equipment purchases, staff training, and technical assistance have alleviated many of these concerns. However, site visits conducted during the implementation phase suggest that between 8% and 16% of CYS children require meal modifications for numerous reasons (e.g., allergies, religious accommodations). This CYS figure is consistent with the estimated prevalence of childhood food allergies in the United States, which is at roughly 8.0% [18,19]. This figure is also consistent with the estimated prevalence of parent-reported childhood food allergies, which, at 11%, is higher owing to the inclusion of reaction histories inconsistent with immunoglobulin E-mediated food allergy [19]. The relatively large proportion of children with special diet requirements adds significantly to the responsibility and workload of the CYS staff, as well as that of other child nutrition professionals. In a survey of child nutrition program menu planners, the majority of respondents agreed or strongly agreed that labor costs were affected by the following factors: (a) purchasing of special food products, (b) planning special diet menus, (c) preparation of food separately to avoid cross-contact, (d) research of suitable substitutions, and (e) additional time for documentation on production records sheets [20].

Preparing meals from scratch also adds to the workload of the food program staff. Many cooks were initially enthusiastic about preparing more foods from scratch, but issues related to scratch cooking methods are a challenge. While there is the intent to make certain that each kitchen is staffed optimally to prepare and serve high-quality, nutritious meals, kitchens are often minimally staffed, making it difficult to assure that staff have enough time for the additional preparation steps scratch cooking requires. 

In addition, observations during a series of site visits, as well as through training attendee feedback, indicated that staffing concerns make it difficult (if not impossible) for the food program employees at some locations to attend training, schedule vacation, conduct inventories, or follow-up with vendor issues. The staffing concerns are exacerbated by the relatively low wages food service staff are paid on average in the U.S. both in the restaurant sector [21] and within the federal government [22]. In fact, federal food service workers are paid one of the lowest average wages of all federal employees, at just over $1600 more per year than a biological science student trainee [22]. When coupled with the demands and responsibilities (e.g., allergen management, food safety, sanitation, and consumer satisfaction) of the childcare food service workers, staff retention is an ongoing concern.

## 6. Conclusions 

In this case report, the process of successfully implementing evidence-based dietary recommendations in a geographically dispersed system with centers worldwide has been described. A comparison of the pre-initiative menus with a current menu indicates that there has been a significant improvement in diet quality. In particular, the post-initiative menu scores reflect an improvement in total diet quality, fatty acid composition, and the offerings of vegetables. Overall, the initiative has demonstrated the ability to further improve diet quality beyond that of baseline federal requirements with adequate support and resourcing. Although further improvements in diet quality are desirable, the improvements identified in this evaluation are notable, in that optimal growth and development during childhood and adolescence are reliant on sound nutrition. 

To build on this evaluation, future assessments of the initiative should include an assessment of dietary intake. Although the CYS system is unique, many of the lessons learned are applicable to non-military childcare facilities. A sample of the Healthy Menu Initiative menus and resources can be found through an online learning system operated via The Ohio State University [5].

## Figures and Tables

**Table 1:** Energy and Healthy Eating Index-2015 scores for pre- and post-Healthy Menu Initiative menus.

Menu	Days (*N* = 70)	Energy (Kcals) Median (Range) ^1^	Healthy Eating Index Score-2015 Mean (SD) ^2^
Pre-Healthy Menu Initiative small installation	20	681.2 (563.7–831.0)	67.8 (7.59) ^a^
Pre-Healthy Menu Initiative large installation	25	643.7 (579.5–838.8)	66.2 (7.26) ^a^
Post-Healthy Menu Initiative all installation	25	674.5 (539.9–789.8)	75.9 (7.37) ^b^

^1^ Mean ranks did not differ (*p* < 0.05), as analyzed by one-way ANOVA of ranks. ^2^ Overall means found to differ significantly (*F*_(2,67)_ = 12.06, *p* ≤ 0.001), as analyzed by one-way ANOVA. Means without a common superscript letter found to differ from one another (*p* < 0.05), as analyzed by Tukey HSD.

**Table 2:** Frequency of daily maximum HEI-2015 component scores among pre-initiative, post initiative, and all menus.

HEI-2015 Component	Maximum Score	Days Achieving Maximum Score		
		Pre-Healthy Menu Initiative (*n* = 45)	Post-Healthy Menu Initiative (*n* = 25)	All Menus Combined (*N* = 70)
Total Fruits	5	95.6% (*n* = 43)	100% (*n* = 25)	97.1% (*n* = 68)
Whole Fruits	5	100% (*n* = 45)	100% (*n* = 25)	100% (*n* = 70)
Total Vegetables	5	4.4% (*n* = 2)	36% (*n* = 9)	15.7% (*n* = 11)
Greens and Beans	5	33.3% (*n* = 15)	64% (*n* = 16)	44.3% (*n* = 31)
Whole Grains	10	0% (*n* = 0)	4% (*n* = 1)	1.4% (*n* = 1)
Dairy	10	100% (*n* = 45)	100% (*n* = 25)	100% (*n* = 70)
Total Protein Foods	5	35.6% (*n* = 16)	44% (*n* = 11)	38.6% (*n* = 27)
Seafood and Plant Proteins	5	13.3% (*n* = 6)	32% (*n* = 8)	20% (*n* = 14)
Fatty Acids	10	2.2% (*n* = 1)	0% (*n* = 0)	1.4% (*n* = 1)
Refined Grains	10	97.8% (*n* = 44)	100% (*n* = 25)	98.6% (*n* = 69)
Sodium	10	46.7% (*n* = 21)	40% (*n* = 10)	44.3% (*n* = 31)
Added Sugars	10	75.6% (*n* = 34)	92% (*n* = 23)	81.4% (*n* = 57)
Saturated Fats	10	28.9% (*n* = 13)	48% (*n* = 12)	35.7% (*n* = 25)

**Table 3:** Comparison of pre-initiative and post initiative HEI-2015 component scores.

	Median Score (Range)		U Statistic	*p*
HEI-2015 Component	Pre-Healthy Menu Initiative (*n* = 45)	Post-Healthy Menu Initiative (*n* = 25)		
Total Fruit	5.0 (4.8–5.0)	5.0 (5.0–5.0)	537.50	0.288
Whole Fruit	5.0 (5.0–5.0)	5.0 (5.0–5.0)	562.50	1.0
Total Vegetables	1.8 (1.4–5.0)	4.6 (3.0–5.0)	90.00	<0.001
Greens and Beans	0.0 (0.0–5.0)	5.0 (0.0–5.0)	398.00	0.025
Whole Grains	0.0 (0.0–5.2)	0.0 (0.0–10.0)	509.00	0.444
Dairy	10.0 (10.0–10.0)	10.0 (10.0–10.0)	562.5	1.0
Total Protein Foods	4.8 (0.0–5.0)	4.9 (0.9–5.0)	483.00	0.316
Seafood and Plant Proteins	0.0 (0.0–5.0)	0.0 (0.0–5.0)	435.00	0.067
Fatty Acids	0.7 (0.0–10.0)	2.8 (0.0–8.4)	356.00	0.010
Refined Grains	10.0 (9.5–10.0)	10.0 (10.0–10.0)	550.00	0.456
Sodium	9.0 (1.0–10.0)	9.0 (4.0–10.0)	530.00	0.676
Added Sugars	10.0 (4.5–10.0)	10.0 (7.0–10.0)	470.50	0.096
Saturated Fats	7.2 (2.4–10.0)	9.0 (3.0–10.0)	376.50	0.190

## Supplementary Materials

The following are available online at https://www.mdpi.com/1660-4601/17/8/2746/s1, Supplement 1: Pilot Menu Evaluation Tool, Supplement 2: Menu Spring 2019.
