# Supplementary material for: A Model for Improving Diet Quality within Child Nutrition Programs: The U.S. Army’s Child and Youth Services Healthy Menu Initiative
Source: Int J Environ Res Public Health. 2020 Apr 16;17(8):2746. doi: 10.3390/ijerph17082746 (PMC7216194; doi:10.3390/ijerph17082746)
Supplement: Supplementary file 1 [file ijerph-17-02746-s001.zip › Supplement_1 Pilot Menu Evaluation Tool.pdf]

	Beef Paprikash	Red Cabbage	Chicken Alfredo with a twist	Sweet potato plum bread square	Taco pie with beans	Porcupine sliders	Whole grain cinnamon nachos	Strawberries & blueberries
Effort Required to Prepare Meal (Use Preparation Scale.)	4	2	3	3	3	4	3	3
Time it Took to Prepare Meal (Use Preparation Scale.)	3	2	3	3	3	4	3	3
Quality of ingredients provided by vendor? (Use Quality Scale.) Explain.	3	3	3	3	3	3	3	3
Quality (taste, textures, visual appeal) of finished product? (Use Quality Scale.) Explain.	3	3	5	3	3	3	4	4
Did This Meal Require any Special Equipment or Kitchen Skills?<br>Yes or No<br>If yes, what?	NO	YES, cutting up large amounts of cabbage	NO	YES, large mixer	NO	NO	NO	NO
General Comments/Suggestions for the day	The kids did not eat it based on looks.<br><br>Sour cream could be reduced. It was a little overpowering.	The kids didn't even try it.<br><br>None of the children liked it.	The kids loved it.<br><br>All children like this.<br><br>This is an obvious favorite.	The kids and staff for the most part loved it.<br><br>Taste was good. Nice change for the children.<br><br>Very good cake not to sweet and soft.	Staff liked it but the kids didn't.<br><br>Easy make.<br><br>It was a hit with the children!	The kids love these, its just the time to prepare them.<br><br>It was a hit with the children!	Very popular with the kids.<br><br>The whole grain tortillas are chewy when baked.	Very popular with the kids.<br><br>These fruits are out of season and the kids eat the blueberries but not the strawberries.
Should We Make This Meal Again? (Yes, maybe, or no.)	Maybe	No	Yes	MAYBE	YES, I think they will learn to like it	YES	YES	YES
	Preparation Difficulty Scale<br>1 2 3 4 5<br>Less difficult or less time — Similar to usual — More difficult or more time				Quality Scale<br>1 2 3 4 5<br>Poor Quality — Similar to usual — Excellent Quality			
