# Supplementary material for: A Model for Improving Diet Quality within Child Nutrition Programs: The U.S. Army’s Child and Youth Services Healthy Menu Initiative
Source: Int J Environ Res Public Health. 2020 Apr 16;17(8):2746. doi: 10.3390/ijerph17082746 (PMC7216194; doi:10.3390/ijerph17082746)
Supplement: Supplementary file 1 [file ijerph-17-02746-s001.zip › Supplement_2 Menu Spring 2019.pdf]

Installation/Facility____ Week of/Dates:____

Week 1*	Minimum Serving Size Per Age							
	1-2	3-5	6-18	**Monday**	**Tuesday**	**Wednesday**	**Thursday**	**Friday**
**Breakfast**								
Fluid Milk**	1/2 c	3/4 c	1 c	Fluid Milk 1% or Whole	Fluid Milk 1% or Whole	Fluid Milk 1% or Whole	Fluid Milk 1% or Whole	Fluid Milk 1% or Whole
Fruit/ Veg	1/4 c	1/2 c	1/2 c	Kiwi***	Hash brown potatoes	Honeydew melon***	Banana	Orange (Mandarin oranges)
Bread/ Grain	1/2 serving	1/2 serving	1 serving	WGR Rice Chex	WGR toast	WGR toasted oat cereal	WGR fruit & bran muffin	WGR waffle
Meat/ Other				Yogurt	Scrambled eggs			Pancake syrup (optional)
**Lunch**				Meatloaf with WGR parsley noodles	Tuna salad with WGR crackers	Baked chicken	BBQ pork slider***	Cheesy rice casserole
Meat/ Alt	1 oz	1 1/2 oz	2 oz	Ground beef	Tuna	Chicken	Pork	Egg, cheese
Bread/ Grain	1/2 serving	1/2 serving	1 serving	WGR pasta	WGR crackers	Quinoa pilaf***	WGR roll	Brown rice
Fruit	1/8 c	1/4 c	1/4 c	Carrots	Banana	Collard greens***	Coleslaw*** (cauliflower)	Pineapple (crushed pineapple)
Vegetable	1/8 c	1/4 c	1/2 c	Green beans	Broccoli (steamed for CDC)	Sweet potatoes	Pinto beans	Peas
Fluid Milk**	1/2 c	3/4 c	1 c	Fluid Milk 1% or Whole	Fluid Milk 1% or Whole	Fluid Milk 1% or Whole	Fluid Milk 1% or Whole	Fluid Milk 1% or Whole
Other					Creamy vegetable dip^^			
**PM Snack**								
Fruit	1/2 c	1/2 c	3/4 c	Apple	Orange (Mandarin oranges)	Pear	Peaches	
Vegetable	1/2 c	1/2 c	3/4 c					Broccoli & cauliflower (steamed for CDC)
Meat/ Alt	1/2 oz	1/2 oz	1 oz	Cheddar cheese (sliced)			Yogurt	
Bread/ Grain	1/2 serving	1/2 serving	1 serving	WGR soft pretzel	Trail mix [no pretzels or dried fruit for CDC]^	Graham crackers without honey		WGR crackers
Fluid Milk**	1/2 c	1/2 c	1 c		Fluid Milk 1% or Whole	Fluid Milk 1% or Whole		Fluid Milk 1% or Whole
Other						Sunbutter^^	Granola^^	Creamy vegetable dip

*All hard foods must be cut to 1/4 inch for children under 2 and to 1/2 inch for children 2-3 years of age. For children under 3 years of age replace menu item with the food in parenthesis. **For children under 18 months, see appendix for important information.** Foods will not be cut onto rounds in CYS Facilities. Water available at meals/snacks and in classroom. Condiments are served as appropriate. WGR is whole grain rich. Menu subject to change. **Serve only unflavored whole milk to children age one. Serve only unflavored 1% or skim milk to children 2 years old and older. *** See menu notes for additional information or substitution options. ^Cooking project. ^^SAC only. Additional serving size information is located in the weekly notes and appendix.

This institution is an equal opportunity provider.

Approved by: ____ Signature Date:____

XXXXX, Nutritionist IMCOM G9 Menus approved for use through: ________________________

Installation/Facility____ Week of/Dates:____

Week 1 Notes

Monday

- ***Kiwi
  May substitute pears for kiwi

- Yogurt
  1-5 years: 1/4 cup
  6-18 years: 1/2 cup

- Meatloaf
  1-2 years: 1/2 piece
  3-5 years: 3/4 piece
  6-18 years: 1 piece

- Parsley noodles
  1-5 years: 1/4 cup
  6-18 years: 1/2 cup

Tuesday

- Scrambled eggs
  1-5 years: 2 Tbsp
  6-18 years: 1/4 cup

  Tuna salad
  1-2 years: 1/4 cup
  3-5 years: 3/8 cup
  6-18 years: 1/2 cup

- Creamy vegetable dip
  1-5 years: none
  6-18 years: 1 1/2 Tbsp

- Trail mix:
  1-5 years: 5/8 cup (no pretzels or dried fruit)
  6-18 years: 1 cup (made with pretzels) or 1 1/8 cup (made without pretzels)

Wednesday

- ***Honeydew melon
  May substitute apples for honeydew melon

- ***Quinoa pilaf
  May substitute bulgur for quinoa, see quinoa pilaf recipe

- ***Collard greens
  May substitute turnip greens, sautéed spinach, kale, or chard for collard greens

Thursday

- BBQ pork sliders
  1-2 years: 1/2 sandwich
  3-18 years: 1 sandwich

- BBQ pork sliders***
  May substitute sloppy lentil Joes for BBQ pork sliders

Friday

- Cheesy rice casserole
  1-5 years: 1/4 cup
  6-18 years: 1/2 cup

Approved by: ____ Signature Date:____

XXXXX, Nutritionist IMCOM G9 Menus approved for use through: _________________________

Installation/Facility____ Week of/Dates:____

Week 2*	Minimum Serving Size Per Age							
	1-2	3-5	6-18	**Monday**	**Tuesday**	**Wednesday**	**Thursday**	**Friday**
**Breakfast**								
Fluid Milk**	1/2 c	3/4 c	1 c	Fluid Milk 1% or Whole	Fluid Milk 1% or Whole	Fluid Milk 1% or Whole	Fluid Milk 1% or Whole	Fluid Milk 1% or Whole
Fruit/ Veg	1/4 c	1/2 c	1/2 c	Pears	Blueberries***	Peaches***	Pineapple (crushed pineapple)	Cantaloupe
Bread/ Grain	1/2 serving	1/2 serving	1 serving	WGR corn puffs cereal (KIX)	Oatmeal	WGR French toast bake	WGR bagel	Bran flakes (WGR Life***)
Meat/ Other						Yogurt	Scrambled eggs	
**Lunch**				Bean burrito bowl	Chicken Alfredo***	Shepard's pie***	Lemon baked fish	Turkey burger slider
Meat/ Alt	1 oz	1 1/2 oz	2 oz	Pinto beans & cheese	Chicken	Beef	Fish	Ground turkey
Bread/ Grain	1/2 serving	1/2 serving	1 serving	Cilantro brown rice	WGR pasta	WGR biscuit	WGR breadstick	WGR roll
Fruit	1/8 c	1/4 c	1/4 c	Peaches	Pineapple (crushed pineapple)	Orange (Mandarin oranges)	Broccoli	Green beans
Vegetable	1/8 c	1/4 c	1/2 c	Diced tomatoes, romaine lettuce	Spinach salad (sautéed spinach)	Potatoes, carrots, peas [in pie]	Black beans	Potato wedges
Fluid Milk**	1/2 c	3/4 c	1 c	Fluid Milk 1% or Whole	Fluid Milk 1% or Whole	Fluid Milk 1% or Whole	Fluid Milk 1% or Whole	Fluid Milk 1% or Whole
Other								Lettuce, tomato, mayonnaise
**PM Snack**								
Fruit	1/2 c	1/2 c	3/4 c			Apples	Banana	Pear
Vegetable	1/2 c	1/2 c	3/4 c	Parmesan roasted cauliflower***, marinara sauce	Cherry tomato & corn salad***^ + carrots (steamed)	Cinnamon sweet potato cubes		
Meat/ Alt	1/2 oz	1/2 oz	1 oz					Monterey-Jack cheese (sliced)
Bread/ Grain	1/2 serving	1/2 serving	1 serving		WGR pita bread		Graham crackers without honey	WGR crackers
Fluid Milk**	1/2 c	1/2 c	1 c	Fluid Milk 1% or Whole		Fluid Milk 1% or Whole	Fluid Milk 1% or Whole	
Other					Hummus		Sunbutter^^	

*All hard foods must be cut to 1/4 inch for children under 2 and to 1/2 inch for children 2-3 years of age. For children under 3 years of age replace menu item with the food in parenthesis. **For children under 18 months, see appendix for important information.** Foods will not be cut onto rounds in CYS Facilities. Water available at meals/snacks and in classroom. Condiments are served as appropriate. WGR is whole grain rich. Menu subject to change. **Serve only unflavored whole milk to children age one. Serve only unflavored 1% or skim milk to children 2 years old and older. *** See menu notes for additional information or substitution options. ^Cooking project. ^^SAC only. Additional serving size information is located in the weekly notes and appendix.

This institution is an equal opportunity provider.

Approved by: ____ Signature Date:_______________________________________

XXXXX, Nutritionist IMCOM G9 Menus approved for use through: _________________________

Installation/Facility____ Week of/Dates:____

Week 2 notes

Monday

- Bean burrito bowl
  1-2 years: 1/4 c rice, 1/3 cup bean mixture, 1/8 cup tomatoes, (no lettuce for this age group), 2 tsp shredded cheese
  3-5 years: 1/4 c rice, 1/2 cup bean mixture, 1/8 cup tomatoes, 1/4 cup shredded lettuce, 1Tbsp shredded cheese
  6-18 years: 1/2 c rice, 2/3 cup beans, 1/4 cup tomatoes, 1/2 cup shredded lettuce, 1 Tbsp plus 1 tsp shredded cheese.

- *** Parmesan roasted cauliflower
  For children under 18 months old serve steamed cauliflower with Parmesan cheese sprinkled on top old

- Parmesan roasted cauliflower:
  1-5 years: 3/8 cup
  6-18 years: 1/2 cup

- Marinara sauce:
  1-5 years: 1/8 cup
  6-18 years: 1/4 cup

Tuesday

- *** Blueberries
  For children under 18 months chopped or pureed

- ***Chicken Alfredo
  May serve meat and pasta separately (see optional recipe)

- Chicken Alfredo
  1-2 years: 1/2 cup
  3-5 years: 3/4 cup
  6-18 years: 1 cup

- ***Cherry tomato & corn salad
  For children under 18 months old omitted this dish and replace with equal amount of carrots.

- Cherry tomato and corn Salad
  1-2: none
  2-5 years: 1/4 cup
  5-18 years: 3/8 cup

- Carrots
  1-2: 1/2 cup
  2-5 years: 1/4 cup
  5-18 years: 3/8 cup

Wednesday

- ***Peaches
  May substitute mango for peaches

- ***Peas in Shepard's pie
  For children under 18 months old, puree, chop, or omit. If omitted replace with equal amount of carrots.

- Beef Shepard's pie
  1-2 years: 1/2 cup
  3-5 years: 3/4 cup
  6-18 years: 1 cup

Thursday

- Scrambled eggs
  1-5 years: 2 Tbsp
  6-18 years: 1/4 cup

- Lemon baked fish:
  1-2 years: 1/2 portion
  3-5 years: 2/3 portion
  6-18 years: 1 portion

Friday

- WGR Life***
  May substitute WGR Rice Chex for WGR Life

- Turkey burger sliders
  1-2 years: 1/2 slider
  3-18 years: 1 slider

Approved by: ____ Signature Date:_______________________________________

XXXXX, Nutritionist IMCOM G9 Menus approved for use through: _________________________

Installation/Facility_______________________________________ Week of/Dates:_______________________________________

Week 3*	Minimum Serving Size Per Age							
	1-2	3-5	6-18	**Monday**	**Tuesday**	**Wednesday**	**Thursday**	**Friday**
**Breakfast**					Breakfast taco***			
Fluid Milk**	1/2 c	3/4 c	1 c	Fluid Milk 1% or Whole	Fluid Milk 1% or Whole	Fluid Milk 1% or Whole	Fluid Milk 1% or Whole	Fluid Milk 1% or Whole
Fruit/ Veg	1/4 c	1/2 c	1/2 c	Peaches***	Pineapple (crushed pineapple)	Banana	Strawberries	Orange (Mandarin oranges)
Bread/ Grain	1/2 serving	1/2 serving	1 serving	WGR English muffin	WGR tortilla	WGR toasted oat cereal	Oven-baked whole wheat pancake	WGR Rice Chex
Meat/ Other					Scrambled eggs	Yogurt	Pancake syrup (optional)	
**Lunch**				Cheesy noodles with beef***	Roasted pork ***	Teriyaki turkey	Southwestern white bean soup	Crispy baked chicken***
Meat/ Alt	1 oz	1 1/2 oz	2 oz	Ground beef & cheese	Pork***	Turkey	White beans + cheddar cheese (sliced)	Chicken
Bread/ Grain	1/2 serving	1/2 serving	1 serving	WGR pasta	WGR roll	Brown rice	WGR breadstick	WGR biscuit
Fruit	1/8 c	1/4 c	1/4 c	Cauliflower	Broccoli	Pears	Apple	Collard greens
Vegetable	1/8 c	1/4 c	1/2 c	Carrots	Black beans	Peas	Green beans	Sweet potatoes
Fluid Milk**	1/2 c	3/4 c	1 c	Fluid Milk 1% or Whole	Fluid Milk 1% or Whole	Fluid Milk 1% or Whole	Fluid Milk 1% or Whole	Fluid Milk 1% or Whole
Other								
**PM Snack**						Lemon pasta salad***^		
Fruit	1/2 c	1/2 c	3/4 c	Pear	Apple			Peaches
Vegetable	1/2 c	1/2 c	3/4 c			Tomato, cucumber + broccoli [steamed for CDC]	Potato wedges & marinara sauce	
Meat/ Alt	1/2 oz	1/2 oz	1 oz					Yogurt
Bread/ Grain	1/2 serving	1/2 serving	1 serving	Trail mix [no pretzels or dried fruit for CDC]^	Graham Crackers without honey	WGR pasta		
Fluid Milk**	1/2 c	1/2 c	1 c	Fluid Milk 1% or Whole	Fluid Milk 1% or Whole		Fluid Milk 1% or Whole	
Other					Sunbutter^^	Creamy vegetable dip		Granola^^

*All hard foods must be cut to 1/4 inch for children under 2 and to 1/2 inch for children 2-3 years of age. For children under 3 years of age replace menu item with the food in parenthesis. For children under 18 months, see appendix for important information. Foods will not be cut onto rounds in CYS Facilities. Water available at meals/snacks and in classroom. Condiments are served as appropriate. WGR is whole grain rich. Menu subject to change. **Serve only unflavored whole milk to children age one. Serve only unflavored 1% or skim milk to children 2 years old and older. *** See menu notes for additional information or substitution options. ^Cooking project. ^^SAC only. Additional serving size information is located in the weekly notes and appendix.

This institution is an equal opportunity provider.

Approved by: ____ Signature Date:_______________________________________

XXXXX, Nutritionist IMCOM G9 Menus approved for use through: _________________________

Installation/Facility____ Week of/Dates:____

Week 3 Notes

Monday

- ***Peaches
  May substitute mango for peaches

- *** Cheesy noodles with beef
  May serve meat and pasta separately (see optional recipe)

- Cheesy noodles with beef
  1-2 years: ½ c + 1 tsp
  3-5 years: ¾ c + 1/2 Tbsp
  6-18 years: 1 c+ 1 Tbsp

- Trail mix:
  1-5 years: 5/8 cup (no pretzels or dried fruit)
  6-18 years: 1 cup (made with pretzels) or 1 1/8 cup (made without pretzels)

Tuesday

- ***Corn in breakfast tacos
  Omit for CDC

- Breakfast Taco:
  1-5 years: 1 taco
  6-18 years: 1 taco

- *** Roasted pork
  May substitute chicken for roasted pork

Wednesday

- Yogurt
  1-5 years: 1/4 cup
  6-18 years: 1/2 cup

- Turkey Teriyaki:
  1-2 years: 1/2 serving
  3-5 years 3/4 serving
  6-18 years: 1 serving

- ***Cucumbers in lemon pasta salad:
  For children under 18 months old, remove skin on cucumbers

- Lemon Pasta Salad:
  1-5 years: 1/2 cup
  6-18 years: 1 cup

- Broccoli
  1-18 years: 1/4 cup

Thursday

- ***White beans and corn in southwestern white bean
  For children under 18 months old, chop corn and beans or puree soup.

- Southwestern white bean soup
  1-2 years: 1/2 cup
  3-5 years: 1/2 cup
  6-18 years: 1 cup

- Cheddar cheese
  1-2 years: 1/2 oz
  3-5 years: 1 oz
  6-18 years: 1 oz

- Potato Wedges:
  1-5 years: 3/8 cup
  6-18 years: 1/2 cup

- Marinara:
  1-5 years: 1/8 cup
  6-18 years: 1/4 cup

Friday

- ***Crispy baked chicken
  For children under 18 months, serve chicken without breading.

Approved by: ____ Signature Date:____

XXXXX, Nutritionist IMCOM G9 Menus approved for use through: __________________________

Installation/Facility____ Week of/Dates:________________________________

Week 4*	Minimum Serving Size Per Age							
	1-2	3-5	6-18	Monday	Tuesday	Wednesday	Thursday	Friday
**Breakfast**								
Fluid Milk**	1/2 c	3/4 c	1 c	Fluid Milk 1% or Whole	Fluid Milk 1% or Whole	Fluid Milk 1% or Whole	Fluid Milk 1% or Whole	Fluid Milk 1% or Whole
Fruit/ Veg	1/4 c	1/2 c	1/2 c	Banana	Applesauce	Pineapple (crushed pineapple)	Blueberries	Orange (Mandarin orange)
Bread/ Grain	1/2 serving	1/2 serving	1 serving	Bran flakes (WGR Life***)	WGR biscuit	Oatmeal	WGR waffle	WGR bagel
Meat/ Other					Scrambled eggs		Pancake syrup (optional)	Yogurt
**Lunch**				Roasted turkey	Lemon salmon cake***	Frittata	Chicken curry	Spaghetti & meat sauce
Meat/ Alt	1 oz	1 1/2 oz	2 oz	Turkey	Salmon	Egg	Chicken	Ground beef
Bread/ Grain	1/2 serving	1/2 serving	1 serving	WGR bread dressing	WGR roll	WGR breadstick***	Brown rice	WGR pasta
Fruit	1/8 c	1/4 c	1/4 c	Broccoli	Coleslaw (carrots)	Cantaloupe***	Pear	Peaches
Vegetable	1/8 c	1/4 c	1/2 c	Pinto beans	Potato wedges	Peas	Green beans	Tossed salad (broccoli)
Fluid Milk**	1/2 c	3/4 c	1 c	Fluid Milk 1% or Whole	Fluid Milk 1% or Whole	Fluid Milk 1% or Whole	Fluid Milk 1% or Whole	Fluid Milk 1% or Whole
Other								Italian salad dressing
**PM Snack**							English muffin pizza^	
Fruit	1/2 c	1/2 c	3/4 c	Honeydew melon***	Orange (Mandarin orange)	Apple		
Vegetable	1/2 c	1/2 c	3/4 c			Cinnamon sweet potato cubes	Tomato sauce, vegetable toppings + carrots (steamed for CDC)	Cucumber and cauliflower (steamed for CDC)
Meat/ Alt	1/2 oz	1/2 oz	1 oz				Cheese on pizza	
Bread/ Grain	1/2 serving	1/2 serving	1 serving	Carrot muffin***	Trail mix		WGR English muffin	WGR crackers
Fluid Milk**	1/2 c	1/2 c	1 c	Fluid Milk 1% or Whole	Fluid Milk 1% or Whole	Fluid Milk 1% or Whole		
Other							Creamy vegetable dip	Sesame dipping sauce

*All hard foods must be cut to 1/4 inch for children under 2 and to 1/2 inch for children 2-3 years of age. For children under 3 years of age replace menu item with the food in parenthesis. For children under 18 months, see appendix for important information. Foods will not be cut onto rounds in CYS Facilities. Water available at meals/snacks and in classroom. Condiments are served as appropriate. WGR is whole grain rich. Menu subject to change. **Serve only unflavored whole milk to children age one. Serve only unflavored 1% or skim milk to children 2 years old and older. *** See menu notes for additional information or substitution options. ^Cooking project. ^^SAC only. Additional serving size information is located in the weekly notes and appendix.

This institution is an equal opportunity provider.

Approved by: ____________________________________ Signature Date:________________________________

XXXXX, Nutritionist IMCOM G9 Menus approved for use through: ________________________

Installation/Facility____ Week of/Dates:____

Week 4 Notes

Monday

- WGR Life***
  May substitute WGR toasted oat cereal for WGR Life cereal

- WGR bread dressing
  1-5 years: 1/2 piece
  6-18 years: 1 piece

- ***Honeydew melon
  May substitute crushed pineapple for honeydew melon

- ***WGR carrot muffins: two recipe options for WGR carrot muffin. One is with quinoa and the other is with oatmeal.

- Carrot muffin
  1-5 years: 1/2 muffin
  6-18 years: 1 muffin

Tuesday

- Scrambled eggs
  1-5 years: 2 Tbsp
  6-18 years: 1/4 cup

- ***Lemon baked salmon cake
  May substitute tuna for salmon in recipe

- Lemon baked salmon cakes:
  1-2 years: 2/3 cake
  3-5 years: 1 cake
  6-18 years: 1 1/3 cake (or one large) see recipe

- Trail mix:
  1-5 years: 5/8 cup (no pretzels or dried fruit)
  6-18 years: 1 cup (made with pretzels) or 1 1/8 cup (made without pretzels)

Wednesday

- Frittata
  1-2 years: 1/2 piece
  3-5 years: 3/4 piece
  6-18 years: 1 piece

- ***Breadstick
  May substitute WGR toast for breadstick

- ***Cantaloupe
  May substitute banana for cantaloupe

Thursday

- Chicken curry
  1-2 years: 1/2 serving (about 3 Tbsp)
  3-5 years: 3/4 serving (about 1/4 cup)
  6-18 years: 1 serving (about 1/3 cup)

- English muffin pizza
  1-5 years: 1 pizza
  6-18 years: 2 pizzas

- Carrots
  1-5 years: 1/2 cup
  6-18 years: 5/8 cup

Friday

- Meat spaghetti sauce
  1-2 years: 1/4 cup
  3-5 years: 3/8 cup
  6-18 years: 1/2 cup

- Sesame dipping sauce
  1-5: 1 Tbsp
  6-18 years: 1 1/2 Tbsp

Approved by: ____ Signature Date:_______________________________________

XXXXX, Nutritionist IMCOM G9 Menus approved for use through: _________________________

Installation/Facility____ Week of/Dates:____

Week 5*	Minimum Serving Size Per Age							
	1-2	3-5	6-18	**Monday**	**Tuesday**	**Wednesday**	**Thursday**	**Friday**
**Breakfast**					Breakfast tacos***			
Fluid Milk**	1/2 c	3/4 c	1 c	Fluid Milk 1% or Whole	Fluid Milk 1% or Whole	Fluid Milk 1% or Whole	Fluid Milk 1% or Whole	Fluid Milk 1% or Whole
Fruit/ Veg	1/4 c	1/2 c	1/2 c	Orange (Mandarin oranges)	Applesauce	Kiwi***	Cantaloupe	Peaches
Bread/ Grain	1/2 serving	1/2 serving	1 serving	Toasted oat cereal	WGR tortilla	WGR bagel	WGR French toast bake	WGR corn puffs cereal KIX
Meat/ Other					Scrambled eggs	Cream cheese	Yogurt	
**Lunch**				Bean enchilada bake	Roasted pork***	Strawberry*** spinach salad with chicken	Sesame beef	Turkey sandwich
Meat/ Alt	1 oz	1 1/2 oz	2 oz	Beans & cheese	Pork	Chicken	Beef	Turkey
Bread/ Grain	1/2 serving	1/2 serving	1 serving	WGR tortilla***	WGR pasta	WGR crackers	Brown rice	WGR bread
Fruit	1/8 c	1/4 c	1/4 c	Peaches	Carrots	Apple	Pineapple (crushed pineapple)	Cucumber, celery^^
Vegetable	1/8 c	1/4 c	1/2 c	Tossed salad (green beans)	Black beans	Spinach (sautéed)	Sautéed cabbage (peas)	Potato, corn & cauliflower soup***
Fluid Milk**	1/2 c	3/4 c	1 c	Fluid Milk 1% or Whole	Fluid Milk 1% or Whole	Fluid Milk 1% or Whole	Fluid Milk 1% or Whole	Fluid Milk 1% or Whole
Other				Salad dressing				Creamy vegetable dip
**PM Snack**								Cinnamon nachos^
Fruit	1/2 c	1/2 c	3/4 c		Pear			Mango*** and bananas
Vegetable	1/2 c	1/2 c	3/4 c	Broccoli & cauliflower (steamed for CDC)		Potato wedges & marinara sauce	Carrots [steamed for CDC]	
Meat/ Alt	1/2 oz	1/2 oz	1 oz		Cheese			
Bread/ Grain	1/2 serving	1/2 serving	1 serving	WGR soft pretzel	WGR crackers		WGR pita bread	WGR tortilla
Fluid Milk**	1/2 c	1/2 c	1 c	Fluid Milk 1% or Whole		Fluid Milk 1% or Whole		
Other				Creamy vegetable dip			Hummus	

*All hard foods must be cut to 1/4 inch for children under 2 and to 1/2 inch for children 2-3 years of age. For children under 3 years of age replace menu item with the food in parenthesis. For children under 18 months, see appendix for important information. Foods will not be cut onto rounds in CYS Facilities. Water available at meals/snacks and in classroom. Condiments are served as appropriate. WGR is whole grain rich. Menu subject to change. **Serve only unflavored whole milk to children age one. Serve only unflavored 1% or skim milk to children 2 years old and older. *** See menu notes for additional information or substitution options. ^Cooking project. ^^SAC only. Additional serving size information is located in the weekly notes and appendix.

This institution is an equal opportunity provider.

Week 5 Notes

Approved by: ______________________________________ Signature Date:________________________________

XXXXX, Nutritionist IMCOM G9 Menus approved for use through: ________________________

Installation/Facility____ Week of/Dates:____

Monday

- Bean enchilada bake
  1-2 years: 1/2 cup
  3-5 years: 3/4 cup
  6-18 years: 1 cup

- ***WGR tortilla
  May use WGR flour or WGR corn tortilla

Tuesday

- ***Corn in breakfast tacos
  Omit for CDC

- Breakfast Taco:
  1-5 years: 1 taco
  6-18 years: 1 taco

- Roasted pork***
  May substitute pinto beans for roasted pork. If this substitution is made then add broccoli as the vegetable.

Wednesday

- ***Kiwi
  May substitute crushed pineapple for kiwi

- Strawberry spinach salad
- 1-2 years: none
  3-5 years: 1/2 cup
  6-18 years: 1 cup

- ***Strawberries
  May substitute mandarin oranges for strawberries

- Potato wedges:
  1-5 years: 3/8 cup
  6-18 years: 1/2 cup

- Marinara:
  1-5 years: 1/8 cup
  6-18 years: 1/4 cup

Thursday

- Yogurt
  1-5 years: 1/4 cup
  6-18 years: 1/2 cup

- Sesame beef
  1-2 years: 1/2 serving (1 oz cooked)
  3-5 years: 3/4 serving (1 1/2 oz cooked)
  6-18 years: 1 serving (2 oz cooked)

Friday

- *** Potato, corn, and cauliflower soup
  For children under 18 months old, puree corn or substitute cauliflower for soup.

- Potato, corn, and cauliflower soup:
  1-2 years: 1/4 cup
  3-5 years: 1/2 cup
  6-18 years: 1 cup

- Turkey Sandwich
  1-2 years: 2 quarter pieces of sandwich
  3-5 years: 3 quarter pieces of sandwich
  6-18 years: 1 full sandwich

- Creamy vegetable dip
  1-5 years: 1 Tbsp
  6-18 years: 1 1/2 Tbsp

- Mango***
  May substitute strawberries for mango.

- WGR cinnamon nachos (with 6-inch tortilla)
  1-5 years: 1/2 tortilla plus 1/2 c fruit
  6-18 years: 1 tortilla plus 3/4 c fruit

- WGR cinnamon nachos (with 8-inch tortilla)
  1-5 years: 1/3 tortilla plus 1/2 c fruit
  6-18 years: 2/3 tortilla plus 3/4 c fruit

Approved by: ____ Signature Date:_______________________________________

XXXXX, Nutritionist IMCOM G9 Menus approved for use through: _________________________

Installation/Facility____ Week of/Dates:____

Appendix:

Unless noted otherwise in the menu template or weekly notes, serving sizes are as listed below.

½ serving of bread/grains = ½ slice of bread; ¼ cup of cooked breakfast cereal, cooked rice, cooked pasta or cooked grains; 4 saltine crackers; 1 whole graham cracker sheet (2 ½ x 5 inches); ½ muffin, ½ serving of muffin squares, cornbread, or French toast bake; ½ of a 1-oz biscuit, roll, or soft pretzel; ½ of a mini-bagel; ¼ of a 2-oz bagel; ½ of a 1.25-oz waffle; ¼ of a 2-oz English muffin or pita.

1 serving of bread/grains = 1 slice of bread; ½ cup of cooked breakfast cereal, cooked rice, cooked pasta or cooked grains; 8 saltine crackers, 2 whole graham cracker sheets (2 ½ x 5 inches); 1 muffin, 1 serving of muffin squares, cornbread, or French toast bake; 1 1-oz biscuit, roll, or soft pretzel; 1 mini bagel, ½ of a 2-oz bagel; 1 1.25-oz waffle; and ½ of a 2-oz English muffin or pita.

The serving size for ready to eat breakfast cereal varies by type:

WGR Rice Chex or WGR Life	WGR corn puffs cereal	WGR toasted oat cereal	Bran flakes
1-2 years: ½ cup	1-2 years: ¾ cup	1-2 years: ½ cup	1-2 years: ½ cup
3-5 years: ½ cup	3-5 years: ¾ cup	3-5 years: ½ cup	3-5 years: ½ cup
6-18 years: 1 cup	6-18 years: 1 ¼ cup	6-18 years: 1 cup	6-18 years: 1 cup

Ounce equivalents for meats/ alternates: cooked meat and cheese, 1 oz = 1 oz; cooked beans/ peas, ¼ c = 1 oz; yogurt, 4 oz (½ c) = 1 oz,

For "other" foods that do not count as a meal component, **unless specified otherwise in the weekly notes**, the serving size is below:

Hummus	Creamy vegetable dip	Sunbutter	Granola
1-5 years: 2 Tbsp	1-5 years: 1 ½ Tbsp	1-5 years: none	1-5 years: 0
6-18 years: 3 Tbsp	6-18 years: 2 Tbsp	6-18 years: 1 Tbsp	6-18 years: 2 Tbsp

Cream Cheese: Spread thin to prevent choking hazard, about ½ tsp per ½ serving of bagel (i.e., ½ tsp on ½ of a mini-bagel or ¼ of a 2-oz bagel).

Pancake Syrup: 2 tsp per ½ waffle or ½ serving of pancake bake. No syrup on French toast bake.

Salad Dressing: 1 ½ tsp per ½ cup of salad or 1 Tbsp per cup of salad.

Smart Balance: ½ tsp per ½ serving of toast, bagel, English muffin, brown bread, roll, and cornbread. ½ tsp per ¼ cup mashed potatoes. No spread on biscuits calculated, but may be used.

Approved by: ____ Signature Date:____

XXXXX, Nutritionist IMCOM G9 Menus approved for use through: _________________________

Installation/Facility____ Week of/Dates:____

For children under 18 months, the following foods should be modified as noted or a substitution should be offered.

Apples: Offer applesauce.

Blueberries: Should be chopped or pureed.

Cheese: Should be served shredded or sliced in thin pieces.

Crispy baked chicken: Serve chicken without breading.

Coleslaw: Should not be given to this age group. Offer a substitution.

Corn: Puree, chop, or offer a substitution.

Cucumber: Should be served without skin, may require steaming if not soft enough to be cut with fork.

Crushed pineapple: Should not be given to this age group. Offer a substitution.

Fish: Serve tuna or checked carefully for bones.

Grapes: Even when cut into quarters, grapes should not be given to this age group. Offer a substitution.

Parmesan roasted cauliflower: Serve steamed cauliflower with Parmesan cheese sprinkled on top.

Peas: Should be chopped or pureed.

Southwestern white bean soup: Chop corn and beans or puree soup.

Approved by: ____ Signature Date:____

XXXXX, Nutritionist IMCOM G9 Menus approved for use through: __________________________
